# Technologies for Detection of *Babesia microti*: Advances and Challenges

**DOI:** 10.3390/pathogens10121563

**Published:** 2021-11-30

**Authors:** Scott Meredith, Miranda Oakley, Sanjai Kumar

**Affiliations:** Laboratory of Emerging Pathogens, Division of Emerging and Transfusion-Transmitted Diseases, Office of Blood Research and Review, Food and Drug Administration, Silver Spring, MD 20993, USA; Scott.Meredith@fda.hhs.gov (S.M.); Miranda.Oakley@fda.hhs.gov (M.O.)

**Keywords:** *Babesia microti*, antibody-based assays, nucleic acid tests, multiplex detection, next generation sequencing

## Abstract

The biology of intraerythrocytic *Babesia* parasites presents unique challenges for the diagnosis of human babesiosis. Antibody-based assays are highly sensitive but fail to detect early stage *Babesia* infections prior to seroconversion (window period) and cannot distinguish between an active infection and a previously resolved infection. On the other hand, nucleic acid-based tests (NAT) may lack the sensitivity to detect window cases when parasite burden is below detection limits and asymptomatic low-grade infections. Recent technological advances have improved the sensitivity, specificity and high throughput of NAT and the antibody-based detection of *Babesia*. Some of these advances include genomics approaches for the identification of novel high-copy-number targets for NAT and immunodominant antigens for superior antigen and antibody-based assays for *Babesia*. Future advances would also rely on next generation sequencing and CRISPR technology to improve *Babesia* detection. This review article will discuss the historical perspective and current status of technologies for the detection of *Babesia microti*, the most common *Babesia* species causing human babesiosis in the United States, and their implications for early diagnosis of acute babesiosis, blood safety and surveillance studies to monitor areas of expansion and emergence and spread of *Babesia* species and their genetic variants in the United States and globally.

## 1. Introduction

*Babesia microti* is an intraerythrocytic, apicomplexan parasite that is the primary agent responsible for human babesiosis in the United States. *B. microti* is transmitted sporadically in many temperate regions of the world, but its prevalence is highest in New England and the northern Midwest region of the United States [1,2,3,4]. In spite of its global transmission and public health impact, *B. microti* infections often remain undetected, resulting in undiagnosed or delayed diagnosis of acute babesiosis cases, which could be fatal in vulnerable individuals, and asymptomatic chronic infections, which present a risk to blood safety. At the time of the discovery of the parasites responsible for babesiosis in livestock in 1888, diagnosis relied on the then-new technology of microscopic examination of stained blood films [5]. The first case of human babesiosis was identified in 1957, and an outbreak on Nantucket Island established the disease in the United States [6,7,8,9,10,11]. Since this time, disease prevalence has increased sharply from only a few cases a year to as many as 2418 in the United States in 2019 [12]. Detection technologies have improved accordingly, but the biology of *B. microti*. and its infection kinetics in humans present unique challenges that have not been fully met by any one detection technology.

Advancements in the technologies for *B. microti* detection have improved diagnostic capability, primarily by making the reliable identification of low-grade early infections for clinical diagnosis possible and the monitoring of treatment. In addition, superior assay sensitivity provides a valuable tool to identify blood donors with chronic, low-grade infections, as the *B. microti* parasite is one of the most commonly transfusion-transmitted pathogens in United States. In this review article, we discuss the advances in detection methods for *B. microti*, in the context of clinical diagnosis, epidemiology and molecular surveillance, and blood safety since the discovery of the parasite over 130 years ago. The field of *Plasmodium* parasite detection and epidemiology is more advanced compared to *Babesia*. Given the biological similarities and detection challenges, we have drawn parallels and applied lessons from this pathogen throughout the article.

## 2. *Babesia microti* Biology and Detection Challenges

The dynamics of the natural course of human babesial infection has still yet to be fully defined in absence of a human challenge model. Our knowledge of the kinetics of infection and parasite burden mostly comes from observations in clinical and epidemiological studies and data from asymptomatic blood donors in endemic areas. From a clinical standpoint, it is assumed that most symptomatic babesiosis cases develop within 1 to 4 weeks following exposure, but clinicians are recommended to consider babesial infection in patients with tick bites within the previous six months [13]. Furthermore, tick bites are often unnoticed, which, in addition to complicating diagnosis, makes extrapolation of the incubation period difficult [13]. Transfusion-transmitted babesiosis (TTB) cases provide a more definitive timepoint for acquisition of the infection, though patients are not monitored for infection early after transfusion before the onset of symptoms. Nevertheless, in these cases, evidence suggests that the incubation period of *B. microti* prior to the appearance of illness ranges from one to nine weeks in most cases, though one patient did not develop symptoms until six months after transfusion of infected blood product [14].

**Figure 1:**
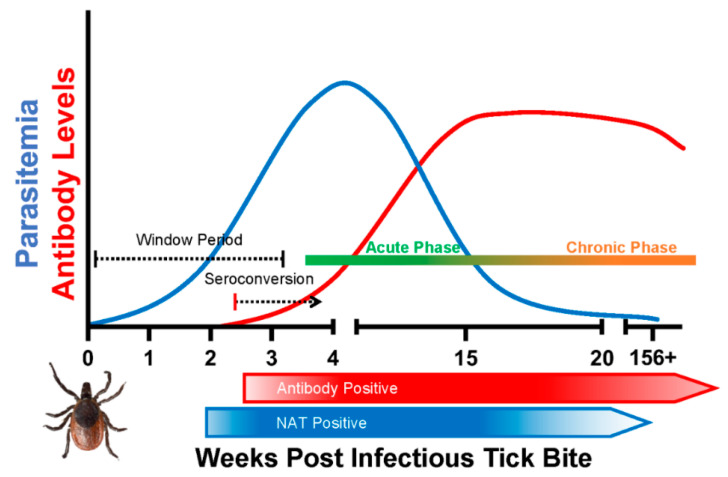
A schematic of course of *Babesia microti* infection and induction and duration of antibody response after an infectious tick bite in a healthy human host. The time frame for the window period (time to infectious bite to first detection of parasitemia), acute phase and chronic phase of infection are based on the observations from clinical cases, epidemiological studies and follow up studies in transfusion-transmitted infections.

The intraerythrocytic nature of *B. microti* infection presents several challenges to effective detection of the parasite. Blood film microscopy and xenodiagnosis are the most direct methods for *Babesia* diagnosis. Parasite nucleic acid and antigen detection are considered the reliable biomarkers of active infection, whereas antibodies can be indicative of active infection or a previously resolved infection. After a tick bite or transfusion of infected blood product, parasitemia remains below detectable limit during the early phase of infection (window period), followed by a relatively higher parasitemia (acute) phase and finally a persistent (chronic) infection in some individuals (Figure 1).

Experimental infection of rhesus monkeys is the only available data describing early *B. microti* infection kinetics in primates. Intravenous inoculation resulted in parasitemia detectable by microscopy in seven of eight monkeys after prepatent periods ranging from 15 to 46 days [15]. On the other hand, infection transmitted via tick bite was established in four of five monkeys after a prepatent period of 13 to 28 days [16]. In these studies, blood film microscopy was the only tool employed to determine the early infection, and it is not known when molecular assays would have become effective.

In humans, parasite burdens higher than 1% are commonly observed in acute severe babesiosis patients. A review of 139 human babesiosis cases requiring hospitalization found a significant correlation between disease severity and parasitemia ≥ 4% [17]. While high parasitemias are a useful marker in identifying risk of severe outcomes, severe cases can develop in individuals with lower parasitemias. Another case series of 34 patients with babesiosis requiring hospitalization found the median parasitemia in these severe cases to be 7.6%, though individuals ranged from 0.1% to 30%; anemia was more strongly correlated with severe outcomes than parasitemia [18]. Parasitemia has been observed as high as 85% in an asplenic individual [19].

The proportion of *Babesia* infections that persist as asymptomatic, chronic infections is not clearly known. In one study on Block Island (Rhode Island), one-third of *Babesia* infections were asymptomatic [20], although the sample size was too small to draw firm conclusions. In addition, in endemic areas, the contribution of reinfection to chronic parasitemia has not been investigated. Nonetheless, results from limited clinical observations and donor screening for *Babesia* by nucleic acid-based test (NAT) assays are beginning to shed some light on the duration of the persistence of *B. microti* infections in asymptomatic individuals living in endemic areas. In one case report, *B. microti* infection, based on polymerase chain reaction (PCR) results, may persist for up to 27 months without overt clinical illness [21]. However, in an investigational study on blood donors in endemic areas, in the majority of NAT-positive donors the parasitemic period was reported to last from 2–7 months by a PCR-based test [22]. In contrast to the acute parasitemic phase, parasite burden during persistent chronic infection phase is significantly lower, but wide ranging and generally not detectable by microscopy. Results from one study (based on extrapolations from a laboratory-based PCR assay) showed the presence of 5 parasites to 3 million parasites per mL in asymptomatic blood donors in endemic areas [22].

The detection of early infection prior to seroconversion (window period cases) is even more challenging and important for early diagnosis and treatment, particularly in vulnerable population groups. Thus, a combination of detection biomarkers and further technological advances would be required for early diagnosis, epidemiology and to monitor the genetic diversity and geographical spread of human babesiosis in the United States and globally.

## 3. Detection Techniques

**Figure 2:**
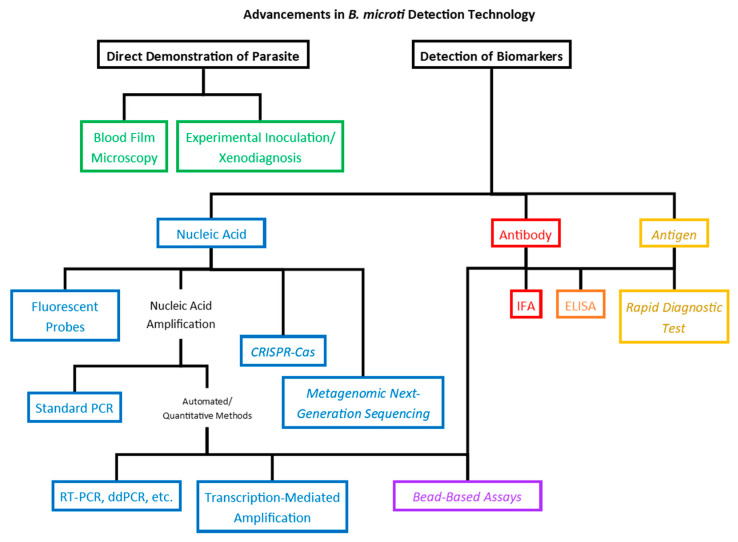
Diagram depicting developments in *B. microti* detection technology. Detection of biomarkers of infection are classified according to the type of biomarker: nucleic acid (blue), antibody (red), or antigen (yellow). Bead-based methods (purple) can be adapted for detection of either nucleic acid or antibody, while ELISA (orange) can be used to detect antibody or antigen. Technologies in italics have been developed for other pathogens and are proposed for detection of *B. microti* but have not yet been effectively adapted.

In the near century and a half since the identification of the parasites that would come to populate the *Babesia* genus, the methods used for detection of the parasites in blood samples have, obviously, become much more sensitive. This became especially true in the latter half of the 20th century as human babesiosis emerged from a medical curiosity to a true public health threat. Modern molecular techniques are at least 10- to 20-fold more sensitive compared to those used at the time of the identification of the parasites, and new technologies on the horizon promise to increase sensitivity while optimizing time and resource economy, providing adaptable platforms with the possibility of multiplexing and possibly elucidating correlations between biomarkers and clinical status or outcomes (Figure 2).

### 3.1. Direct Demonstration of B. microti Parasites

#### 3.1.1. Experimental Inoculation

Xenodiagnosis and experimental inoculation have long been tools for the diagnosis of babesial infection. Babes employed Robert Koch’s third postulate, requiring reproduction of the disease upon inoculation into a healthy, susceptible host in his initial identification of *Babesia* parasites. He did not observe clinical signs in inoculated cattle or other large livestock, though he did see significant disease in inoculated rabbits [5]. Hamsters have been used for the experimental inoculation of *B. microti*, while gerbils, splenectomized calves, and SCID mice are used when appropriate for other *Babesia* species [23,24]. Direct observation of parasites on a blood film is generally easier and much less time consuming for all but the lowest parasitemia cases, as the inoculation of susceptible animal models will take 7–10 days before appreciable amplification of the parasites can be detected. In addition, procuring animals for each diagnosis is significantly more expensive and resource-intensive. Nevertheless, experimental inoculation was a common alternate diagnostic technique for low-parasitemia cases for nearly 100 years until the advent of highly sensitive serological and molecular procedures for *Babesia* detection [24,25].

#### 3.1.2. Blood Film Microscopy

**Figure 3:**
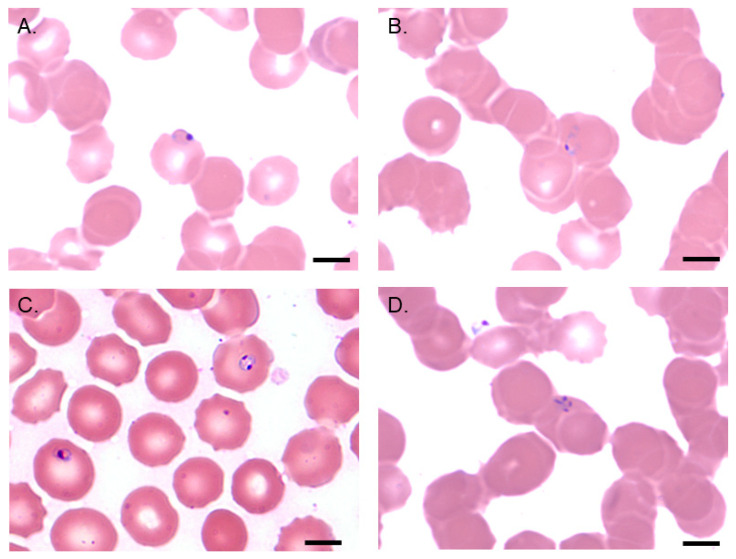
Images from a Giemsa-stained blood film from a human infected with *B. microti*. Trophozoites appear in (**A**) ring forms or in (**B**) mature, amoeboid forms. Merozoites can be seen as (**C**) a multinucleated body during division or as (**D**) tetrads, called the “Maltese Cross” form, following two rounds of division. Scale bar represents 5 μm.

The discovery of the *Babesia* parasite in 1888 by Babes was slightly preceded by the identification of many other blood-borne pathogens, most notably the human malaria parasite in 1880 by Alphonse Levaran [26]. On a Giemsa-stained blood film, the abundant *B. microti* ring-like trophozoites resemble those of *P. falciparum*, though *Babesia* spp. rings tend to be larger with more variation in size and shape. Additionally, *Babesia* spp. rings do not contain pigment and may be vacuolated. Trophozoites divide by binary fission, usually twice, producing a cruciform merozoite structure known as a tetrad or “Maltese Cross” (Figure 3). This form is rare but is pathognomic of *Babesia* infection [27].

In the case of malaria, the limit of detection of a thick blood smear is approximately 10–50 parasites/μL (at least 0.0002% parasitemia), while a thin blood smear alone is roughly 20–40 times less sensitive even under ideal conditions [28]. Furthermore, it has been estimated that the practical limit of detection in a routine diagnostic screen is closer to 100 parasites/μL (0.002% parasitemia) [29,30]. The limit of detection by microscopy has not been determined for *Babesia*. However, similarities in parasite morphology and the blood film preparation method indicate a limit of detection comparable to *Plasmodium* detection by microscopy. Therefore, even if a potential *B. microti*-infected sample is screened with a thick smear and confirmed by thin smear, the risk of failing to identify parasites in prepatent or convalescent stages or among asymptomatic carriers is large. Parasitemia is frequently less than 1%, especially early in infection when treatment is often sought [31], so while blood films can be valuable for confirmation of diagnoses in a low-volume setting, higher-throughput and more sensitive techniques are needed to meet the need presented by the emergence of *B. microti* as a public health threat.

### 3.2. Detection of Biomarkers of B. microti Infection

#### 3.2.1. Nucleic Acid-Based Assays

Since the early 1990s, methods for molecular detection based on the PCR amplification of *B. microti* genes have been tested and validated [24,32,33,34]. The results have shown that molecular methods provide a superior option for the detection of *B. microti* than blood film microscopy. Notably, the increased sensitivity of molecular detection methods has improved the detection of low-grade early infections (window period cases) and chronic infections [22,35]. While complete data are not available for *Babesia* spp. detection, the World Health Organization Methods Manual for the evaluation of *P. falciparum* blood smears indicates that approximately 0.333 μL of blood is screened in a standard thick smear, yielding a limit of detection of roughly 20 parasites per μL [36]. By comparison, even early PCR protocols for *P. falciparum* detection could reliably detect 20 times fewer parasites from a 10-fold smaller sample volume [37]. In addition, molecular methods are better suited for the species differentiation of different *Babesia* strains, as differentiation by microscopy can be difficult or impossible [38].

Recent advances in *B. microti* genomics and detection technologies have led to the development of assays of higher sensitivity and high-throughput platforms for diagnosis, molecular surveillance and blood safety. The adaptation of real-time PCR technology to *B. microti* detection increased the sensitivity dramatically over standard PCR techniques [33]; an early diagnostic RT-PCR protocol exhibited a limit of detection that was roughly ten-fold lower than standard PCR [39,40].

The sample volume used in the assay is another major consideration that determines the sensitivity and limit of detection of an assay. While Persing et al. detected roughly 3 parasites in a 50 μL standard PCR reaction, Bloch et al. calculated a limit of detection of 12.92 parasites per 2 mL of blood by their RT-PCR protocol [33,40]. This equates to roughly 0.39 parasites per 50 μL, which emphasizes the potential impact of sampling error on the sensitivity of molecular detection methods; the procedure may be extraordinarily sensitive, but the sample size may be too small to contain target nucleic acid.

The droplet digital PCR platform has been adapted to *B. microti* detection and achieves sensitivity and limits of detection comparable to RT-PCR [40,41]. In addition, transcription-mediated amplification has been employed in an FDA-licensed detection assay for *B. microti* and has a 95% detection limit of approximately 3 parasites per mL [35].

The *B. microti* genome sequence was first published in 2012 [42]. Analyses revealed a genome of approximately 6.5 Mbp encoding around 500 polypeptides, which is the smallest of all Apicomplexan nuclear genomes [42]. A combination of genomics-based antigen discovery and computational sequence analyses have allowed for the identification of novel high-copy-number conserved detection targets, which was previously not available [42,43,44]. For example, the 18S ribosomal RNA gene is the most commonly used amplification target for *Babesia* spp. detection [33,35,41]. Recently, high-copy-number BMN family genes were evaluated for analytical sensitivity by RT-PCR. In this study, the 18S rRNA gene produced a limit of detection of 30.9 parasites per mL, while the BMN primer set detected as few as 10.0 parasites per mL [45]. Table 1 summarizes the sensitivity of blood film microscopy, experimental inoculation and nucleic acid-based detection of *Babesia* parasites in blood.

To better understand genetic diversity and evolutionary relationships, investigators have begun to sequence the *B. microti* genome from parasite isolates collected from around the world. In one comprehensive study, the complete genome sequencing of 42 *B. microti* samples from different parts of the world showed extensive genetic diversity [48]. As anticipated, *B. microti* samples from the continental US are genetically distant from samples from Alaska, Russia and Japan. In the U.S., deep genetic divergence was noted between samples from the Northeast and the Midwest. Minimal genetic diversity was noted among the New England samples, though three sub-populations exist: Nantucket, mainland New England and the R1 reference group [48]. A study based on a 32 single nucleotide polymorphism (SNP) barcode assay supported previous findings and identified two distinct lineages among the New England and Midwestern *B. microti* parasites [49]. SNP-based barcode assays developed from genome-wide sequencing of recently circulating *B. microti* isolates could be an important surveillance tool to monitor genetic diversity in clinical cases and in the expanding areas of transmission.

It is anticipated that novel high-copy-number conserved gene targets identified by genome analyses, multiplexing for simultaneous detection of *Babesia* species and sub-populations circulating in an area and technological advances including detection target enrichment in a sample would further improve the sensitivity, specificity and applicability of *B. microti* NAT assays for diagnosis, surveillance and blood safety purposes.

#### 3.2.2. Antigen Detection Assays

Antigens expressed by an invading pathogen serve as a reliable biomarker to detect an active infection for many pathogens. Antigen-based rapid detection tests (RDT) are a mainstay of malaria diagnosis in endemic areas. No laboratory-based or commercial RDT for the diagnosis of *B. microti* is available, though potential biomarkers of infection have been identified. In 2000, Lodes et al. screened *B. microti* antigens for immunoreactivity in serological tests [50]; Homer et al. later verified the antigenicity of several novel antigens with the aim of supporting the development of a diagnostic assay [51]. *B. microti* alpha-helical cell surface protein 1 (BmBAHCS1, also known as BmGPI12 [52], BMN1-9 [50] and BmSA1 [53]), a secreted *B. microti* antigen, was identified by Cornillot et al. as the most sensitive antigen for the detection of active infections [52]. Anti-BmBAHCS1 antibodies can be detected in serum as early as 4 (IgM) to 8 (IgG) days following infection in mice, indicating that the detection of the antigen could reduce the window period before the development of a detectable antibody titer [43,52].

Thekkiniath et al. developed an antigen capture assay for the detection of BmBAHCS1 that had a limit of detection of 20 pg/μL in in vitro samples [54]. However, it only identified six of seven clinical samples, failing to detect a sample with a parasitemia of 0.3% [54]. Therefore, further improvements to the assay are required before its widespread adoption. Applications of a combination of genome-wide screening, transcriptional profiling and antigenic characterization in functional assays has led to identification of a large number of immunodominant excreted and secreted and surface-anchored *B. microti* antigens that deserve evaluation as biomarker(s) of active infection [43,44,50,55,56].

Antigen-detection technology is highly advanced for malaria diagnosis. According to the World Health Organization, in 2019, 348 million malaria RDTs were sold globally [57]. The majority of malaria RDTs are based on the plasmodium falciparum histidine-rich protein-2 (PfHRP-2), which is the most reliable marker available for the diagnosis of acute and asymptomatic P. falciparum infections in endemic settings. However, recently, an alarming number of reports indicate deletions of the PfHRP2/PfHRP3 gene and a reduced sensitivity of the HRP-2 based RDTs, thus threatening the effectiveness of HRP-2 based RDTs as a public health tool against malaria [58,59]. These results strongly indicate that *B. microti* antigen-based detection assays should also rely on multiple antigens to offset potential sensitivity loss due emerging polymorphism in target antigens.

In summary, there has been no systematic approach to explore the potential of antigens as biomarkers for diagnosis of human babesiosis. If adequately sensitive and specific, antigen detection-based assays could be an attractive option for the rapid clinical diagnosis and detection of asymptomatic chronic infections in endemic areas.

#### 3.2.3. Antibody-Based Assays

Antibodies are the most sensitive and reliable markers for detection of *Babesia* exposure, albeit with potential limitations in detection in the early phase of infection prior to seroconversion and inability to distinguish between active infection and previously resolved infections. In the early 1970s, several groups began developing indirect immunofluorescence assay (IFA) for the detection of the antibodies indicative of *Babesia* infection in animals, as human babesiosis was rare and considered a curious, if relatively insignificant zoonosis [60,61,62]. By the end of the decade, enough human cases had been identified in residents of Nantucket Island to make the establishment of a standardized protocol for indirect immunofluorescent detection of antibodies directed at *B. microti* antigens necessary and feasible [25,63]. Among antibody-based tests, IFA has been demonstrated to be the most sensitive and detect 100% of blood film-positive acute babesiosis cases and is expected to be highly sensitive in detecting donors with asymptomatic *Babesia* infections, whereas antibody titers are maintained by a low-grade infection [22,25].

However, there has been debate in the literature surrounding the threshold distinguishing active from cleared infections. Chisolm et al. developed the first sensitive and specific technique for the immunofluorescent detection of antibody specific for antigens on the surface of infected erythrocytes and determined that active cases in the acute phase of infection can be loosely defined by a detectable IgG antibody titer of ≥1:1024 [63]. Boustani and Gelfand recommend a titer of ≥1:256 as suggestive of acute infection, while the Centers for Disease Control and Prevention adds that samples from individuals epidemiologically linked to *B. microti* exposure need only exhibit reactivity at a titer of ≥1:64 to be considered a babesiosis case [64,65].

Inter-genus cross-reactivity in indirect immunofluorescence assays is usually low when detecting anti-*B. microti* antibodies, and cross-reactivity with other *Babesia* species is often observed only at lower dilutions [63].

*B. microti*-specific IgG may persist for months or years following infection, which, while valuable for serosurveys that are largely agnostic to the time of infection, could complicate the use of serological tests for diagnostic or donor screening purposes [66]. Ruebush et al. characterized the development of an antibody response to *B. microti* with respect to the onset of symptoms and found that the peak antibody titer was reached around three to four weeks following the onset of symptoms, after which titers decreased over the next several months [67]. The rate of antibody titer decrease was different for each patient and was not correlated with initial antibody titer or severity of illness. One patient was followed for six years after illness and still had an appreciable antibody titer [67]. In a more recent large investigational study, the median time of seroreversion (IFA titer of less than 1:64) in blood donors was 17.1 months [22].

Enzyme-linked immunosorbent assay (ELISA) protocols have been developed for the detection of *B. microti*-specific antibodies using antigens harvested from infected hamsters [68] or mice [69] or using recombinant proteins [44,50,53,70]. The most common antigens exploited by serological assays have been those of the BMN family [50,53]. Historically, compared to IFA, ELISA has been considered less sensitive and specific in detecting acute babesiosis and asymptomatic infections, indicating the need to identify additional immunodominant *B. microti* antigens for use as synthetic peptides or recombinant protein(s) as coating antigens. Recently, a combination of three novel immunodominant *B. microti* antigens (*Babesia microti* Maltese Cross form related protein 1 [BmMCFRP1], *Babesia microti* serine reactive antigen 1 [BmSERA1], and *Babesia microti* piroplasm β-Strand domain 1 [BmPiβS]), when used in combination with the previously described immunodominant antigen BmBAHCS1, yielded 100% sensitivity in the detection of *B. microti*-positive serum samples by ELISA [44].

Thus, it appears that multiple antigens may be needed to achieve the desired sensitivity for an automated alternative to IFA for human babesiosis. Another consideration is the identification of *B. microti* antigens that associated with antibody responses induced during the early phase of infection and could also distinguish between active and resolved infections. To date, no studies have investigated a temporal correlation of parasitemia or clinical condition with titer of antibodies to specific *B. microti* antigens throughout the course of infection. Assays based on such antigens would have a high prognostic value and applications in identifying asymptomatically infected individuals in endemic areas.

## 4. Multiplex Assays

Multiplex assays for *B. microti* have become quite common, as the ability to distinguish between it and other tick-borne diseases in a given area has become more vital. Multiplex PCR assays using standard PCR protocols and fragment size differentiation have been employed for decades [71], but now the RT-PCR platform can easily be adapted to distinguish separate species with the use of fluorescent probes specific for distinct target genes. Historically, these assays have been of particular use in veterinary fields, as livestock and domesticated animals tend to be exposed to a far greater breadth of tick-borne pathogens that need to be distinguished [71,72]. Another application of multiplex PCR assays is for the surveillance of the tick population in a given region to establish the probable rate of exposure to given pathogens [73,74]. RT-PCR techniques routinely detect as few as 10 copies per sample in multiplex assays [74].

Multiplex PCR assays for the detection of tick-borne pathogens in humans are being developed. Buchan et al. evaluated a high definition PCR (HDPCR) panel which contained primers for amplification of target genes of nine species and species groups of tick-borne pathogens [75]. The panel is intended to be an adjunct diagnostic resource for the differentiation of clinical cases suspected to be caused by tick-borne pathogens. The researchers validating the panel observed 100% specificity relative to gold-standard PCR assays for several of the pathogens but did not observe any samples positive for *B. microti* in 530 whole blood specimens, despite high sensitivity among simulated single- and co-infected blood samples. Sensitivity for *Borrelia burgdorferi* was lacking at only 44% relative to standard PCR [75]. It remains to be established whether the lack of detection to *B. microti* is due to performance of the assay or is indicative of the low prevalence of infection among the sample population.

The Luminex bead platform provides an attractive alternative to RT-PCR for multiplex detection assays, as conjugation to xMAP beads allows for the concentration and enhanced differentiation of PCR products. A commercial multiplex bead assay was validated by Livengood et al. for the surveillance of genus- and species-level infection rates of *I. scapularis* ticks [76]. The assay has not been applied to human samples to date. Limits of detection in ticks varied widely across species, but as few as four copies of the *B. microti* target gene (18S rRNA) could be detected [76].

Another application of the multiplex bead assay is the conjugation of recombinantly expressed antigens to spectrally distinct luminescent beads for the detection of antibody specific for each pathogen in a sample. This technique has been applied to differentiation of *B. microti, B. duncani,* and *B. divergens* exposure in human samples [77]. Similar to bead-based PCR techniques, bead-based antibody assays capitalize on the large surface area of the beads for capturing and concentrating the antigen-specific antibody, while relying on species specificity that appears to be characteristic of most human *Babesia* species [76,77,78,79,80,81].

## 5. Novel and Future Technologies

### 5.1. Next Generation Sequencing

Next generation sequencing (NGS) has revolutionized all aspects of medicine. NGS is also being extensively evaluated for the diagnosis and tracking of infectious diseases. The metagenomics NGS (mNGS) is an unbiased approach for the detection of bacteria, fungi, viruses and parasites in clinical samples [82,83,84]. This approach combines the genome sequencing of genetic materials in a biological sample, bioinformatics analysis for exclusion of human reads and pathogen identification based on sequence alignment to a curated database [85]. While mNGS has been successfully applied for pathogen detection including discovery of novel pathogens in clinical samples, sensitivity, specificity and high-cost considerations must be addressed for the routine application of this approach for the routine diagnosis of infection diseases including human babesiosis.

### 5.2. CRISPR Technology

The CRISPR-Cas system is a component of prokaryotic adaptive immunity that protects microbes from invading bacteriophage or plasmid DNA by specifically cleaving foreign genetic elements [86]. In this system, RNA encoding a memorized sequence of foreign DNA “guides” a caspase to a matching target sequence from an invading phage or plasmid that is then destroyed by degradation. Due to its ability to edit genomes, the CRISPR-Cas system has been applied to develop therapeutics to treat genetic diseases. In recent years, the CRISPR-Cas system has also been utilized to develop a new class of rapid, inexpensive, easy-to-use detection systems with high sensitivity and specificity.

The CRISPR-Cas systems of some bacteria contain caspases that collaterally cleave single-stranded nucleic acid in addition to targeting foreign genetic elements. Cas12a and Cas13a (formally C2c2) indiscriminately cleave single-stranded DNA [87] and single-stranded RNA [88], respectively. These systems have been used to develop CRISPR collateral cleavage-based molecular detection platforms where the cleavage of an amplified target pathogen sequence activates collateral cleavage of single-stranded fluorescent or colorimetric reporter molecules. DNA endonuclease-targeted CRISPR trans reporter (DETECTR) [87] and Specific High-Sensitivity Enzymatic Reporter unLOCKing (SHERLOCK) [89] are two diagnostic platforms that use Cas12a and Cas13a to detect DNA and RNA, respectively.

To date, CRISPR-based diagnostics have not been applied to the detection of *Babesia*. However, Cunningham et al. used SHERLOCK CRISPR collateral cleavage-based diagnostics to develop a fast, low-cost deployable assay capable of *Plasmodium* detection, species differentiation and drug-resistance genotyping [90]. This CRISPR-based SHERLOCK assay uses an isothermal RPA reaction to generate double-stranded DNA amplicons of the target sequence, in vitro transcription of RPA product to produce single-stranded RNA (ssRNA) targets and the collateral cleavage of fluorescent or colorimetric RNA reporter molecules to produce a detection signal. When compared to real-time PCR, the *P. falciparum* SHERLOCK assay achieved 94% sensitivity and 94% specificity.

The CRISPR technology warrants the evaluation of species-differentiating detection of human *Babesia* spp. in a high-throughput platform for diagnostic and donor screening purposes.

## 6. Blood Donor Screening

Transfusion-transmitted babesiosis (TTB) is caused by the transfusion of blood and blood products collected from an asymptomatically infected donor. The first case of TTB was reported in 1979 [91]. Since then, more than 250 reported cases of TTB have been reported in the U.S. [10,92,93,94]. Data collected from the national babesiosis surveillance program and other published reports indicate that the clinical burden, areas of transmission and risk to the U.S. blood supply are increasing [10,35,93,94,95]. The intraerythrocytic nature of the parasite and lack of knowledge on minimum parasite burden in the asymptomatic chronic phase of infection present unique challenges in detecting *Babesia* infection in blood donors.

In the past 15 years, laboratory-based NAT and antibody tests have been applied to assess *B. microti* risk in random blood donors in endemic areas. These studies have been useful to gain information on the relative value of NAT and antibodies in identifying asymptomatically infected donors and have shed light on the relationship between seropositivity and parasitemia and the seasonality of transmission in endemic areas [96,97,98]. More recently, two large prospective studies conducted under Investigational New Drug protocols have further enhanced our understanding of the prevalence of *B. microti* infections in asymptomatic healthy blood donors and the rate of window period cases in endemic areas and nonendemic states [22,35,99]. In one investigational study, a total of 89,153 blood donations were screened in four *Babesia* endemic states. Of these, 335 (0.38%) were positive by IFA, and 67 were also PCR positive (20% of IFA+; 0.075% total). A total of nine blood donations were IFA negative but PCR positive (window period cases; 0.01%). Interestingly, 86% of all PCR-positive donors became DNA negative in a one year follow up, while only 8% had seroreversion during the same period, confirming that antibodies continues to persist long after parasitemia clearance [22]. The second investigational study was conducted in 11 endemic states plus Washington D.C. and Florida (nonendemic). Of the 176,926 blood donations initially screened, 61 were confirmed to be positive. Among these samples, 35 (57%; 0.020% of total) were PCR positive and 59 (97%; 0.033% of total) were antibody positive, and 2 (3%; 0.001% of total) were PCR positive but antibody negative (window period cases) [35]. These prospective investigational studies have clearly shown that donor screening for *Babesia* infection allowed for the identification of potentially infectious blood units and thus a valuable tool to minimize the TTB risk to blood supply. Additionally, results complied from the surveillance programs [10,92,93] and investigational studies [22,96] have shown that while tick-borne transmission is seasonal, parasitemic donors can be found year-round. The other finding from these studies indicates that due to travels to endemic areas from nonendemic areas and interstate transport of blood, TTB risk exits outside the outside the bounds of recognized endemic states [100,101].

In May 2019, the FDA issued a guidance document recommending screening blood donors for evidence of *Babesia* infection in 14 high-risk states plus Washington, D.C. through the use of a licensed *Babesia* NAT assay. The effectiveness of regional donor screening for *Babesia* by a licensed NAT assay will be determined based on a significant reduction in the TTB cases in United States.

## 7. Assay Validation

Generally, freshly collected *B. microti* patient samples of known parasite count (by microscopy) are not available for assay validation for diagnosis or blood donor screening. Therefore, the validation of detection assays typically relies on *B. microti* parasites propagated in mice or hamsters and spiked into whole human blood. A reference panel consisting of whole blood spiked with *B. microti* parasites harvested from mice was used to support the licensure of two NAT assays intended for screening blood donations *for B. microti*. By comparison, nucleic acid standards for assay validation of other pathogens, such as Hepatitis C virus (HVC) [102] and human immunodeficiency virus 1 (HIV-1) [103], rely on high-titer isolates from clinical cases or blood product donations. These isolates may be expanded in vitro prior to dilution in human plasma. Efforts should be made to develop validated reference panels based on *B. microti*-infected red blood cells and/or nucleic acid (DNA and RNA) prepared from blood samples from babesiosis patients. Such reference panels should be validated in collaborative studies and made available to assay developers in academia and industry.

## 8. Conclusions

Genomics-based antigen discovery and the incorporation of technological advancements have led to the development of superior NAT and antibody-based assays for human diagnosis. Likewise, the availability of highly sensitive and specific, high-throughput *Babesia* NAT assays have, for the first time, allowed regional donor screening for *Babesia* in endemic states.

Antibody assays based on novel *Babesia* antigens may shorten the window period and allow us to distinguish between acute, persistent chronic and a previously resolved infections.

Antigen-detection based assays in multiplex ELISA format and as RDTs for diagnostics and blood donor screening are awaiting development. It is anticipated that the next generation of assays would also incorporate technological advances offered by mNGS and CRISPR technology.

## Figures and Tables

**Table 1:** Limit of detection for direct observation and molecular methods of detection of *Babesia microti*.

Method	Target	Limit of Detection	Reference
Blood Film		20–100 pRBCs */μL	[28,29,30]
Experimental Inoculation		63 pRBCs/inoculation [into mice]	[46]
Fluorescent Nucleic Acid Probes		100 pg DNA (~30 parasites)[*B. bovis* **]	[47]
PCR	18S rRNA	3 parasites/50 μL	[33]
RT-PCR	18S rRNA	12.92 parasites/2 mL	[40]
	BMN genes	10 pRBCs/mL	[45]
ddPCR	18S rRNA	10 copies	[41]
TMA	18S rRNA	3 pRBCs/mL	[35]

* Parasitized red blood cells. ** No data are available for *B. microti*; this technique was applied for animal *Babesia* species.

## Data Availability

Not applicable.
